# Factor XIII Deficiency: A Review of Biology, Testing, and Treatment

**DOI:** 10.46989/001c.155180

**Published:** 2026-01-20

**Authors:** Jeremy W. Jacobs, Garrett S. Booth, Victoria Costa, Cristina A. Figueroa Villalba, Bipin N. Savani, Brian D. Adkins

**Affiliations:** 1 Department of Pathology, Microbiology, and Immunology, Vanderbilt University, Nashville, TN, USA; 2 Special Coagulation Laboratory, Vanderbilt Medical Laboratories, Nashville, TN, USA; 3 Department of Pathology, NYU Grossman School of Medicine, New York, NY, USA; 4 Department of Laboratory Medicine and Pathology, University of Washington, Seattle, WA, USA; 5 Department of Laboratory Medicine and Pathology, Seattle Children’s Hospital, Seattle, WA, USA; 6 Division of Hematology-Oncology, Department of Medicine, Vanderbilt University Medical Center, Nashville, TN, USA; 7 Division of Transfusion Medicine and Hemostasis, Department of Pathology, University of Texas Southwestern Medical Center, Dallas, TX, USA

**Keywords:** Factor XIII, FXIII, Factor XIII deficiency, Bleeding disorder, Factor deficiency, Cryoprecipitate, Hemostasis, Delayed bleeding, Laboratory testing

## Abstract

Factor XIII (FXIII) deficiency is a rare bleeding disorder characterized by unstable hemostatic clots due to defective fibrin cross‑linking. Congenital FXIII deficiency arises from variants in the F13A1 (FXIII-A subunit) or F13B (FXIII-B subunit) genes, and classically presents with delayed umbilical stump hemorrhage, soft‑tissue and intracranial bleeding, impaired wound healing, and recurrent pregnancy loss. Acquired deficiency stems from inhibitory autoantibodies or from reduced synthesis or consumption in critical illness and surgery. Routine coagulation screening tests are normal and diagnosis relies on quantitative FXIII activity assays with or without antigenic phenotyping and, when indicated, inhibitor testing and molecular confirmation. Plasma‑derived FXIII concentrate reduces spontaneous and intracranial bleeding; recombinant FXIII‑A2 is appropriate for F13A1 defects but not patients with F13B variants. Perioperative and obstetric care target activity thresholds suited to procedural risk and individual pharmacokinetics. This review synthesizes the molecular biology, epidemiology, clinical features, diagnostic methods, and evidence‑based management of FXIII deficiency, with practical guidance for assay selection, validation, and result interpretation.

## 1. Introduction

In 1944, Kenneth Robbins showed that fibrin generated from purified fibrinogen dissolved in weak acid, whereas fibrin formed when serum/plasma was present resisted acid solubilization, implying the existence of a plasma “fibrin stabilizing factor.”^1^ Subsequent work by Laki and Lorand in the 1940s–1950s formalized this concept and produced the classic urea/monochloroacetic acid solubility tests for the factor’s activity.^2^ In 1960, François-Henri Duckert and colleagues reported the first patient with congenital deficiency of this factor in Switzerland; the entity was soon after incorporated into the clotting factor nomenclature as coagulation factor XIII (FXIII), with formal designation adopted in 1963.^3,4^

FXIII is the terminal enzyme of coagulation, activated by thrombin and calcium to catalyze transglutaminase-mediated cross-linking of fibrin γ-chains into dimers and α-chains into high-molecular-weight polymers, while covalently incorporating antifibrinolytic proteins into the nascent clot.^5–8^ These reactions confer tensile strength, viscoelastic stability, and lysis resistance, converting a polymerizing fibrin gel into a durable hemostatic plug.^9^ Because routine screening assays (prothrombin time [PT], activated partial thromboplastin time [aPTT], thrombin time) assess thrombin generation and fibrin formation rather than crosslink maturation, FXIII deficiency presents with normal tests of coagulation despite clinically meaningful impairment of clot stability. Patients may form visually intact clots that fail under physiologic stress or fibrinolytic challenge, yielding delayed hemorrhage and soft tissue and intracranial bleeding.

FXIII deficiency is a rare congenital disorder, most commonly due to variants in the *F13A1* (FXIII-A subunit) or, less often, *F13B* (FXIII-B subunit) genes.^10^ It can also occur as an acquired condition caused by autoantibodies or by consumption/dilution in surgery, trauma, sepsis, and other critical illness.^10^ Diagnostic delays are common given the paradox of normal screening assays and the limited sensitivity of historical clot solubility tests, underscoring the need for validated quantitative FXIII activity methods and inhibitor evaluation when appropriate.^11^ Prophylaxis with FXIII-containing blood products or FXIII concentrates prevents spontaneous and intracranial hemorrhage in congenital disease, and activity-guided replacement may be clinically important in perioperative and obstetric care; nuances surrounding scenarios in which certain replacement products are effective must be considered.^12,13^

This review synthesizes the molecular biology of FXIII, epidemiology and clinical phenotype of congenital and acquired deficiency, contemporary diagnostic strategies (including assay selection, validation, and pitfalls), and evidence-based management across prophylaxis, surgery, obstetrics, and inhibitor states, with practical laboratory and clinical recommendations for hematologists and hemostasis laboratory professionals.

## 2. Biology and Molecular Basis of FXIII

In plasma, FXIII circulates primarily as a non-covalent A_2_B_2_ heterotetramer in which the catalytic A subunits (FXIII-A, encoded by *F13A1*) are chaperoned and stabilized by the carrier B subunits (FXIII-B, encoded by *F13B*).^14–16^ The *F13A1* gene (chromosome 6p24–p25; 15 exons) encodes a 731-amino-acid catalytic subunit that contains the transglutaminase core, while *F13B* (chromosome 1q31–q32.1; 12 exons) encodes a β-propeller/short-consensus-repeat protein composed of 10 sushi domains that confer plasma stability and transport.^17–21^ Deficiency of the FXIII-A subunit is responsible for the majority (~95%) of cases of congenital FXIII deficiency, and typically produces more severe clinical phenotypes due to loss of catalytic activity, whereas FXIII-B subunit defects mainly lower circulating heterotetramer levels by impairing stabilization and carriage.^22–24^

**Figure 1.**
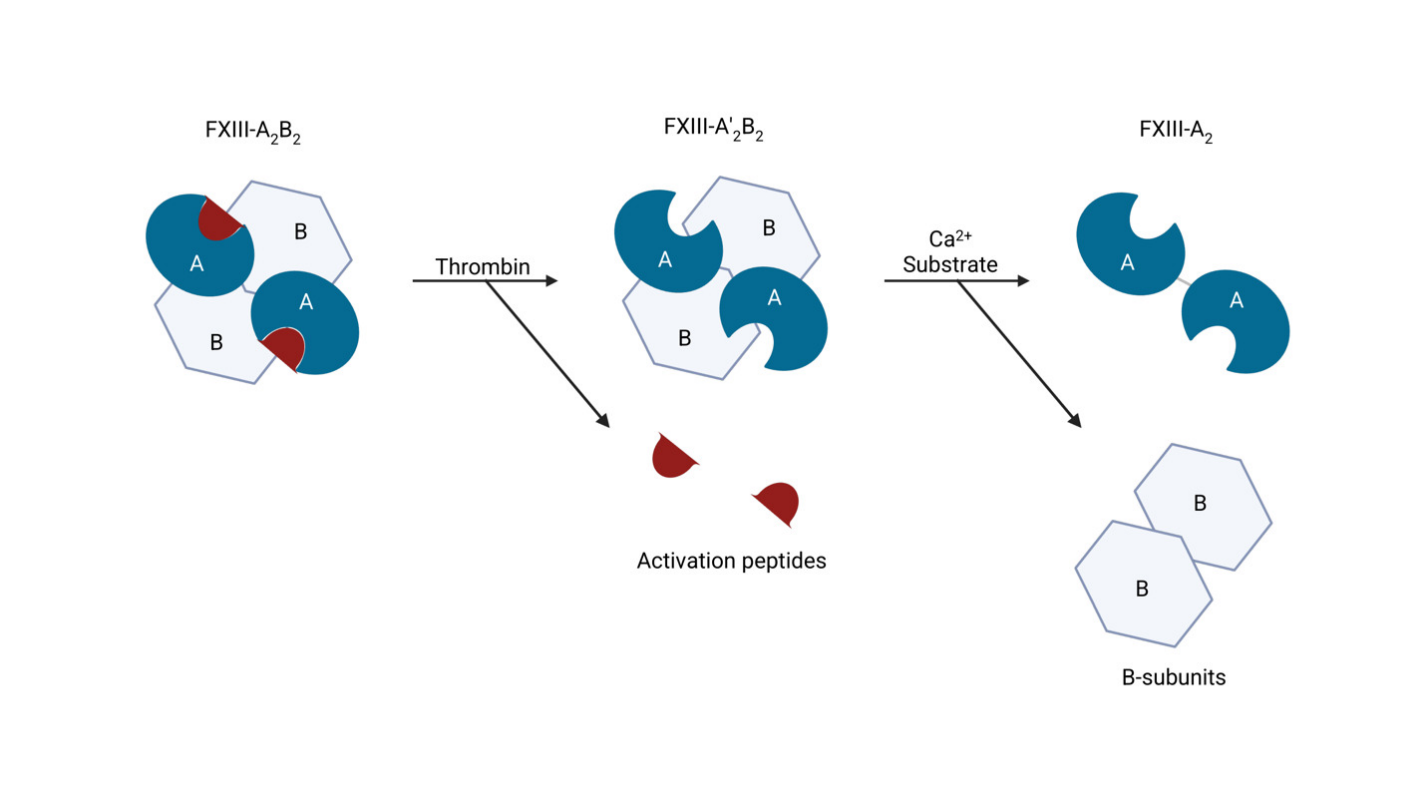
Schematic of factor XIII structure and activation

Activation of zymogen FXIII proceeds in an ordered two-step process (Figure 1). First, thrombin cleaves the N-terminal activation peptide from each FXIII-A subunit, converting the plasma heterotetramer FXIII-A_2_B_2_ to A’_2_B_2_.^25,26^ Second, binding of millimolar Ca^2+^ induces large conformational changes within FXIII-A’_2_ that weaken A–B interactions and release the regulatory FXIII-B_2_ dimer, exposing the catalytic site and yielding the active transglutaminase FXIIIa.^14,27,28^ Structural and kinetic studies support a stepwise, Ca^2+^-facilitated disassembly of the A_2_B_2_ complex during activation.^29,30^

The enzymatic activity is mediated by a conserved Cys-His-Asp triad within the FXIII-A core (Cys314–His373–Asp396).^31^ FXIIIa performs acyl-transfer between γ-carboxamide groups of glutamine donors and ε-amino groups of lysine acceptors to form ε-(γ-glutamyl)-lysine isopeptide bonds.^32^ The best characterized substrates are fibrin chains (γ–γ dimerization and α-chain multimerization), which impart tensile strength and viscoelastic stability to the clot.^33–35^ FXIIIa also crosslinks antifibrinolytic proteins to fibrin; for α_2_-antiplasmin, the predominant linkage involves Gln14 of α_2_-antiplasmin and Lys303 on the fibrin Aα chain, a reaction critical for lysis resistance.^8,36–38^

Beyond fibrin and α_2_-antiplasmin, FXIIIa covalently links fibrin(ogen) to multiple extracellular and plasma proteins (most notably fibronectin and vitronectin), thereby strengthening clot-matrix integration, supporting cell adhesion and angiogenesis, and facilitating wound repair.^39,40^ These matrix-level actions help explain soft tissue bleeding, poor wound healing, and umbilical stump bleeding observed in severe congenital FXIII deficiency.^41–43^

FXIII is also present in cellular pools. Platelets contain abundant FXIII-A, predominantly as an A_2_ dimer without FXIII-B subunits; upon platelet activation, a fraction becomes exposed on the platelet surface and functionally engages within forming thrombi.^9,44,45^ This platelet FXIII-A supports platelet activation dynamics (e.g., spreading/adhesion); loss or genetic deletion of platelet FXIII-A impairs clot retraction and reduces thrombus stability, underscoring distinct roles for cellular (platelet) versus plasma FXIII.^39,46–48^

## 3. Epidemiology and Clinical Phenotype of FXIII Deficiency

### 3.1. Congenital FXIII Deficiency

Congenital FXIII deficiency is a rare inherited bleeding disorder that typically follows autosomal recessive inheritance, with an estimated global prevalence of approximately one in two million individuals (Table 1).^49–52^ The condition shows geographic clustering in regions with higher rates of consanguinity or where founder variants predominate, including parts of Iran, Switzerland, the Middle East, and South Asia.^3,42,49,53–55^

**Table 1.** Concise comparison of congenital and acquired FXIII deficiency

**Domain**	**Congenital FXIII deficiency**	**Acquired FXIII deficiency**
Primary etiology	Biallelic pathogenic variants, predominantly *F13A1* (A-subunit), uncommonly *F13B* (B-subunit) variants; rare heterozygous cases may bleed with additional modifiers	Autoantibodies inhibiting FXIII or non-immune consumption/underproduction (DIC, severe liver disease, major surgery/trauma)
Typical onset	Neonatal/early infancy	Mostly adults; new onset peri-/post-operative or with autoimmune/malignant disease
Sentinel presentations	Delayed umbilical stump bleeding; ICH in infancy; deep soft tissue bleeds; poor wound healing	Disproportionate post-procedural bleeding; soft tissue/retroperitoneal bleeds; mucocutaneous bleeding; potentially life-threatening hemorrhage
Bleeding pattern	Recurrent, major bleeds with severe deficiency	Variable; bleeding may be significant with severe deficiency
Screening coag tests (PT/aPTT/TT)	Typically normal	Typically normal (unless concomitant coagulopathy)
Definitive lab tests	Markedly reduced FXIII activity (often <5–10%); antigen low in A-subunit deficiency and variably low in B-subunit deficiency	Reduced FXIII activity; mixing study shows lack of correction; inhibitor detection/titration where available
Genetics	Identify *F13A1/F13B* variants for confirmation/counseling	No pathogenic variants
Associated conditions	Consanguinity in some cohorts; otherwise isolated	Autoimmune disease, drugs, malignancy; critical illness/consumption
Peri-⁠procedural risk	High without prophylaxis; ICH risk in infancy	High during active inhibitor phase; variable during consumptive states
First-line treatment	FXIII replacement (rFXIII-A2 or pdFXIII) for on-demand and routine prophylaxis; dose to activity targets	Treat bleeding with FXIII concentrate if available; immunosuppression for inhibitor eradication; correct underlying cause in non-immune cases
Bridging when concentrate unavailable	Cryoprecipitate or plasma (variable/low FXIII content) as time-limited bridge	Same bridging for acute control while initiating immunosuppression/definitive therapy
Monitoring	Quantitative FXIII activity for dose tailoring; trough-based prophylaxis; consider PK in pregnancy/pediatrics	Serial FXIII activity and inhibitor titers; monitor response to immunosuppression; reassess need for replacement as inhibitor clears
Point-of-care/assay notes	Prefer quantitative activity assays; viscoelastic tests adjunctive only (not quantitative for FXIII)	Same; add inhibitor detection methods; interpret viscoelastic endpoints cautiously in inhibitor states

Abbreviations: FXIII, factor XIII; rFXIII-A2, recombinant FXIII A-subunit; pdFXIII, plasma-derived FXIII; PT, prothrombin time; aPTT, activated partial thromboplastin time; TT, thrombin time; PK, pharmacokinetics; DIC, disseminated intravascular coagulation; ICH, intracranial hemorrhage

The clinical phenotype varies with residual FXIII activity and is often severe when activity is significantly reduced (≤1-3%). A characteristic neonatal presentation is delayed umbilical stump bleeding, which occurs days to weeks after cord separation in approximately 70-80% of symptomatic cases and serves as an important sentinel feature.^56–58^ In male infants, post-circumcision bleeding is a recognized early presentation that may provide the first diagnostic clue.^59^ The most serious complication is life-threatening intracranial hemorrhage—either spontaneous or following minor trauma—which occurs in up to 30% of untreated patients, often during early childhood.^58^

Beyond the neonatal period, patients with congenital FXIII deficiency commonly experience recurrent mucocutaneous hemorrhage (epistaxis, menorrhagia, oral bleeding), soft-tissue bleeding (e.g., subcutaneous and intramuscular hematomas), and impaired wound repair with dehiscence; abnormal scar formation, including keloids, has been reported in some cases.^12,60^ Joint hemorrhage is relatively uncommon, distinguishing FXIII deficiency from hemophilia.^12^ The impaired wound healing observed in FXIII deficiency reflects multiple mechanisms beyond hemostasis, whereby FXIIIa-mediated crosslinking of fibrin to extracellular matrix proteins creates a stable scaffold essential for fibroblast migration, keratinocyte re-epithelialization, and angiogenesis during tissue repair. Loss of these matrix-stabilizing functions compromises the provisional wound matrix, delays granulation tissue formation, and increases susceptibility to wound dehiscence even when primary hemostasis appears adequate.^61^ Individuals with childbearing potential with severe FXIII deficiency face reproductive challenges, including frequent first trimester pregnancy loss without prophylaxis; the biology represents both impaired hemostasis at the maternal-fetal interface and defective FXIII-dependent stabilization of the extracellular matrix required for successful cytotrophoblast invasion and placental implantation.^62–65^ Notably, while congenital FXIII deficiency is predominantly autosomal recessive, and therefore most patients with a diagnosis of FXIII deficiency carry homozygous or compound heterozygous variants, a subset of individuals with heterozygous FXIII deficiency may still experience bleeding and pregnancy complications.^66^

### 3.2. Acquired FXIII Deficiency

Acquired FXIII deficiency presents across the lifespan through two distinct mechanisms (Table 1). Immune-mediated deficiency develops when autoantibodies—commonly IgG4 subclass—target FXIII-A or, less frequently, FXIII-B subunits.^67–71^ This form typically manifests with the abrupt onset of severe bleeding in previously healthy individuals and may be associated with autoimmune conditions (e.g., systemic lupus erythematosus), malignancy (e.g., non-Hodgkin lymphomas, myeloid neoplasms, colorectal adenocarcinoma and non-small-cell lung cancer), or certain medications (e.g., isoniazid, penicillin, phenytoin), although up to half of cases are idiopathic.^67,68,72–77^

Non-immune acquired deficiency occurs through consumption, dilution, or decreased synthesis of FXIII in critically ill patients.^70,78^ Common precipitants include major surgery (particularly cardiac procedures with cardiopulmonary bypass), severe trauma with hemorrhagic shock, sepsis, liver dysfunction, and disseminated intravascular coagulation.^79–81^ In these settings, FXIII activity can fall to clinically relevant levels, contributing to microvascular bleeding and impaired wound healing.^82^ Although the risk of spontaneous bleeding in congenital deficiency increases below ~10-15% activity, peri-operative and critical care literature suggests aiming for ≥30% (and sometimes higher) to ensure hemostasis.^81,82^

## 4. Diagnostic Approach and Laboratory Methods

**Figure 2.**
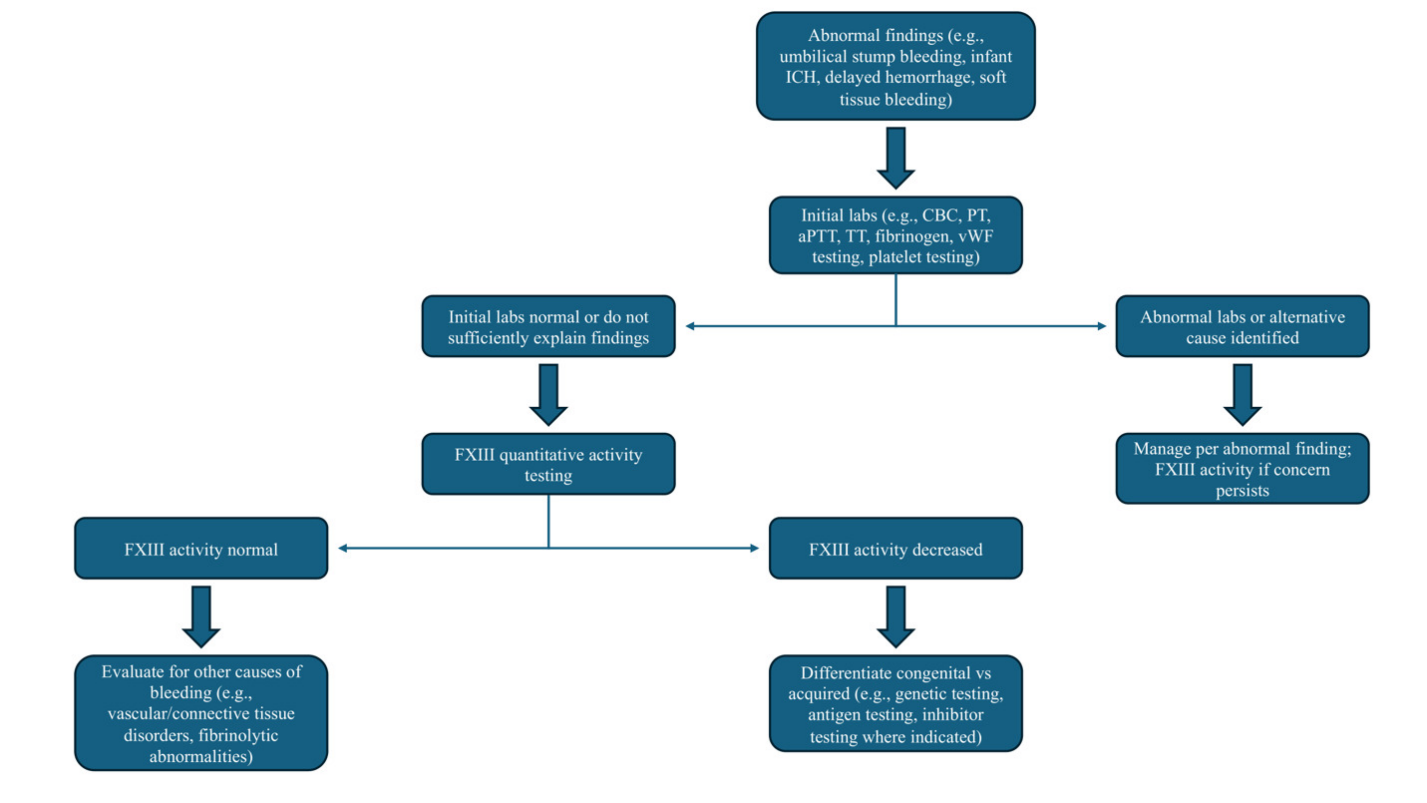
Diagnostic workflow algorithm for suspected factor XIII deficiency

Bleeding that is delayed or out of proportion to the clinical context with normal PT, aPTT, thrombin time, and fibrinogen should evoke suspicion for FXIII deficiency (Figure 2). The plasma sample must be platelet-poor (<10,000/µL), as residual platelets can skew results; given that platelets harbor FXIII-A, platelet contamination can artifactually raise FXIII-A antigen measurements even when soluble plasma FXIII activity is low. As such, guidelines emphasize preparing platelet-poor plasma and measuring both activity and antigen when deficiency is suspected.^83^ Visible hemolysis, lipemia, and marked icterus may interfere with photometric endpoints in some activity methods, and timing relative to replacement therapy must be documented because the extended half-life of FXIII (7-14 days) can mask baseline deficiency if samples are drawn post-dose.^84–86^ For clinical decision-making—especially in prophylaxis titration and perioperative planning—trough sampling immediately before the next scheduled infusion provides the most useful baseline assessment.

### 4.1. Assay Considerations

Classical solubility tests that expose thrombin-generated fibrin clots to 5 M urea or 1% monochloroacetic acid are of limited clinical utility (Table 2). A positive result strongly suggests severe deficiency at very low activity levels (~1 to 5% depending on method); however, sensitivity is poor.^87,88^ Hypofibrinogenemia and dysfibrinogenemia can cause false positives, whereas increased fibrinogen may reduce sensitivity and contribute to false negative results.^89^ Contemporary guidelines therefore discourage reliance on clot solubility assays except as crude adjuncts in low-resource settings; a normal solubility test does not exclude clinically relevant FXIII deficiency and should not delay quantitative FXIII activity testing.^83,90–92^

**Table 2.** Factor XIII laboratory testing

**Assay Type**	**Methodology**	**What It Measures**	**Clinical Utility**	**Limitations**
**Quantitative Activity Assays**				
Ammonia-release	FXIII activated with thrombin/Ca^2+^; liberated NH_3_ measured via glutamate dehydrogenase-coupled NADH oxidation	Enzymatic activity	First-line diagnostic; monitoring prophylaxis and perioperative dosing	Requires specialized equipment; requires a plasma blank (or addition of an FXIII antagonist) to avoid false signal from background ammonia
Isopeptidase	Fluorogenic peptide substrate cleavage (reverse reaction)	Enzymatic activity	Alternative quantitative method; no amine acceptor needed	Less widely available
Amine-incorporation	Chromogenic/fluorogenic detection of labeled amine incorporation into glutamine substrates	Enzymatic activity	Alternative quantitative method	Methodology varies between platforms
**Antigen Assays**				
FXIII-A antigen	Immunoassay (ELISA, immunoturbidimetric)	FXIII-A subunit protein level	Distinguishes quantitative vs qualitative defects; assists in phenotyping F13A1 vs F13B deficiency	Less sensitive for dysfunctional variants; platelet contamination artifacts
FXIII-B antigen	Immunoassay (ELISA, immunoturbidimetric)	FXIII-B subunit protein level	Assists in phenotyping F13A1 vs F13B deficiency	Limited availability
**Functional Screening Tests**				
Clot solubility (5 M urea)	Fibrin clot exposure to 5 M urea; observe dissolution	Cross-linked fibrin stability	Crude screening in low-resource settings	Poor sensitivity (only detects severe deficiency ~1-5%); false positives with hypofibrinogenemia/dysfibrinogenemia
Clot solubility (1% monochloroacetic acid)	Fibrin clot exposure to 1% monochloroacetic acid	Cross-linked fibrin stability	Crude screening in low-resource settings	Same limitations as urea test; not recommended as sole diagnostic
**Inhibitor Testing**				
1:1 Mixing study	Patient plasma mixed 1:1 with pooled normal plasma; measure FXIII activity immediately and after 37°C incubation	Distinguishes deficiency from neutralization	Essential when acquired deficiency suspected	No standardized Bethesda assay for FXIII
Bethesda-style titration	Serial dilutions with FXIII activity measurement	Inhibitor titer (method-specific units)	Quantifies inhibitor strength	Requires adaptation to local FXIII activity method
**Adjunctive Tests**				
Viscoelastic testing (TEG/ROTEM/SEER sonorheometry)	Measure clot formation kinetics and strength	Global hemostasis; late clot firmness	May detect severe deficiency; assess fibrinolysis resistance	Normal results do NOT exclude clinically significant FXIII deficiency; not a substitute for quantitative testing

Abbreviations: FXIII, factor XIII; ELISA, enzyme-linked immunosorbent assay; TEG, Thromboelastography; ROTEM, rotational thromboelastometry; SEER, sonic estimation of elasticity via resonance

Quantitative FXIII activity measurement is the recommended first-line diagnostic modality; while many clinical laboratories still lack the ability to perform such assays, in those that do, at the time of writing, ammonia-release assays predominate.^51,83,85,93^ These assays activate FXIII with thrombin and calcium and then quantify liberated ammonia via a glutamate dehydrogenase–coupled reaction that oxidizes NADH, with the rate of absorbance change proportional to enzymatic activity.^94–96^ Alternative methods include isopeptidase assays, which report FXIIIa activity in reverse by measuring cleavage of a fluorogenic peptide substrate (release of a fluorophore) without requiring an amine acceptor, and amine-incorporation assays, which use chromogenic/fluorogenic readouts to detect incorporation of labeled amines into glutamine-bearing acceptor substrates.^83,84,96^

Antigenic phenotyping adds mechanistic insight once activity is confirmed to be low. In *F13A1*-related quantitative defects, both activity and FXIII-A antigen are reduced, and FXIII-B antigen may be secondarily decreased because heterotetramer formation is impaired. In *F13B* deficiency, FXIII-B antigen is low/absent, while FXIII-A antigen is typically reduced due to loss of stabilization (platelet FXIII-A may be preserved).^9,97^ Antigen testing is less sensitive than activity testing for qualitative (dysfunctional) variants, but it is valuable for distinguishing quantitative defects, informing genotype–phenotype inference, and monitoring reconstitution after infusion.^89,93^

Evaluation for an inhibitor should be performed when low FXIII activity is newly detected in adults or when FXIII activity results are discordant with the reported medical history (i.e., low activity but no history of bleeding). A 1:1 mixing study using patient plasma and pooled normal plasma, with activity measured immediately and after incubation at 37°C for 1-2 h, generally can distinguish deficiency from neutralization.^83,93^ Although there is no universally standardized Bethesda assay for FXIII, laboratories can adapt Bethesda-style inhibitor titration to their validated FXIII activity method and report results in method-specific units while confirming antibody presence with immunoassays to FXIII-A and/or FXIII-B, when available.^93,98^

Viscoelastic testing (VET) (thromboelastography [TEG], rotational thromboelastometry [ROTEM], sonic estimation of elasticity via resonance [SEER] sonorheometry) have important limitations in FXIII deficiency when evaluating bleeding. Standard tracings for ROTEM and TEG primarily reflect thrombin-driven fibrin formation and clot kinetics, while maximum clot strength is dominated by fibrinogen and platelet contribution.^99,100^ Because FXIII-mediated crosslinking primarily augments late clot firmness and resistance to fibrinolysis, isolated FXIII deficiency may yield normal baseline VET parameters, unless the deficit is severe or results are assessed before and after FXIII supplementation.^100^ Experimental protocols that add exogenous tissue plasminogen activator to unmask lysis susceptibility are not standardized, and a normal viscoelastic profile cannot exclude clinically significant FXIII deficiency.^101,102^ Consequently, viscoelastic results should be interpreted as adjunctive and not as substitutes for quantitative FXIII activity in diagnostic or dosing decisions.

Molecular confirmation refines genetic counseling, reproductive planning (carrier testing, prenatal/early neonatal diagnosis, peripartum management), and therapeutic product selection. In practice, targeted next-generation sequencing (NGS) panels that include *F13A1* and *F13B* and incorporate copy-number analysis identify the causative variants in most congenital cases; rare deep intronic or structural variants may require reflex methods.^93,103,104^ Because recombinant FXIII-A_2_ (catridecacog) replaces only the FXIII-A subunit, it is appropriate for *F13A1* deficiency (i.e., the majority of cases) but not for isolated *F13B* deficiency, a distinction that has direct therapeutic implications.^105^

## 5. Management

The central objectives of care are primary prophylaxis to prevent spontaneous and traumatic bleeding, including mitigation of intracranial risk, and targeted replacement during high-risk periods such as surgery and pregnancy.^106^ Because FXIII has a relatively long half-life compared with upstream coagulation factors, scheduled replacement at four-week intervals is typically effective when individualized by activity monitoring and assessment of clinical response.^107^ Plasma-derived FXIII concentrates (pdFXIII; e.g., Corifact/Fibrogammin) remain first-line for routine prophylaxis across age groups and for peri-operative support (Table 3). Product labeling supports an initial dose of 40 IU/kg intravenously every 28 days with subsequent titration (typically in 5 IU/kg increments) to maintain a trough activity of ~5–20%, recognizing interpatient pharmacokinetic variability and the tendency toward higher clearance in some children.^56,107^ Recombinant FXIII-A_2_ (catridecacog; Tretten) is an effective alternative for congenital *F13A1* (FXIII-A subunit) deficiency on a similar every 28-day schedule, but is not indicated for *F13B* (FXIII-B subunit) deficiency; availability can vary by region and formulary and should be confirmed locally.^108^

**Table 3.** Factor XIII replacement products

**Product**	**Indication**	**FXIII per vial/unit (approximate)**	**Final volume**	**FXIII concentration (approximate)**	**Key Considerations**
Plasma-derived FXIII (Corifact/Fibrogammin)	All congenital FXIII deficiency (defects in both *F13A1* and *F13B*)	250 IU (small) or 1,250 IU (large)	4 mL or 20 mL	62.5 IU/mL	First-line for prophylaxis and perioperative use; pathogen-inactivated; availability varies by region
Recombinant FXIII (catridecacog; NovoThirteen/Tretten)	F13A1 (FXIII-A subunit) deficiency only	2,500 IU per vial (NovoThirteen Nominally 2500 IU per vial (2000 – 3125 IU) (Tretten)	3 mL	833 IU/mL (NovoThirteen) 667-1042 IU/mL (Tretten)	NOT indicated for FXIII-B subunit deficiency; pathogen-free; availability varies by region
FFP / PF24	Temporary bridge when concentrates unavailable	288 +/- 77 IU per unit	~200-250 mL	~1-1.5 IU/mL	Variable FXIII content; large volumes; transfusion reactions
Cryoprecipitate	Temporary bridge when concentrates unavailable	~60 ± 30 IU per unit	~15–25 mL	**~**2–4 IU/mL	Variable FXIII content; availability of pathogen-reduction varies

Abbreviations: FXIII, factor XIII; FFP, fresh frozen plasma; PF24, plasma frozen within 24 hours after phlebotomy

On-demand treatment for bleeding and peri-operative management generally employs pdFXIII 20–40 IU/kg IV, followed by FXIII activity assessment and redosing to keep levels above hemostatic targets throughout the risk window. Many centers aim for ≥20–30% activity for minor to moderate procedures and proportionally higher activity targets for major operations, tailored to the institutional assay and practice environment.^107,109,110^ FXIII activity testing is used to confirm recovery and guide interval adjustments to sustain the chosen trough. Long-term prophylaxis with pdFXIII or catridecacog substantially reduces spontaneous bleeding and intracranial events; adverse effects are uncommon, with infrequent hypersensitivity reactions and rare thromboembolic events, while inhibitors are exceedingly rare in congenital disease but should be suspected with poor recovery or unexpectedly short half-life.^107,108,111^

### 5.1. Pediatric Dosing and Monitoring

Children often require closer activity-guided titration because interpatient variability in recovery and clearance can be greater than in adults, particularly in early childhood.^107^ A practical approach is to initiate standard prophylaxis (e.g., pdFXIII every 28 days) and adjust in small increments to maintain individualized troughs suitable for daily activities (commonly ≥5–10%), with higher peri-procedural targets. For on-demand or peri-operative management, weight-based dosing followed by confirmation of recovery and repeat activity testing timed to the clinical risk window may be considered, recognizing that some pediatric patients demonstrate shorter effective half-life and may need earlier redosing to keep activity at hemostatic thresholds. Trough sampling immediately prior to scheduled doses should be considered to capture baseline needs and to ensure an optimal treatment interval or dose as children grow.

### 5.2. Pregnancy

Pregnancy requires anticipatory coordination among hematology, obstetrics, anesthesia, transfusion medicine, and pharmacy. Evidence from case reports, case series, and reviews suggests that a pragmatic approach may be to maintain trough activity of ≥10–20% through early and mid-gestation, escalating to ≥30% activity in late pregnancy and peripartum, with pre-delivery dosing (e.g., 10–40 IU/kg) to support vaginal delivery or cesarean and early postpartum hemostasis, though these are based on observational data/expert opinion.^62,112^ Limited case-based experience suggests neuraxial anesthesia may be able to be performed safely when FXIII activity is maintained at commonly used thresholds (often ≥30% activity) and no additional coagulopathy exists^113,114^; given sparse prospective data, decisions should be individualized with close multidisciplinary planning. Newborns in affected families warrant prompt assessment for congenital deficiency to ensure prophylaxis can be instituted early if indicated.^115^

### 5.3. FXIII Inhibitors

Acquired FXIII inhibitors present unique therapeutic challenges. FXIII concentrates have been used, but their success may vary depending on the titer, target, and kinetics of the autoantibody. Acute hemostasis also relies on supportive measures, and anti-fibrinolytics may also be considered.^67^

Eradication of the inhibitor is the primary long-term goal.^77^ First-line immunosuppression typically combines corticosteroids with or without rituximab; response usually occurs within 4–8 weeks but may require prolonged therapy. Refractory cases may warrant cyclophosphamide, mycophenolate mofetil, or other steroid-sparing agents.^77,116,117^ During immunosuppression, serial FXIII activity and inhibitor titer monitoring (weekly initially, then less frequently as titers decline) guides duration of therapy.^118^ Underlying triggers (e.g., autoimmune disease, malignancy, or offending medications) should be addressed concurrently.^70^ Unlike congenital disease, inhibitor eradication generally allows return to normal hemostasis without ongoing replacement therapy, though relapse can occur and warrants surveillance.^77,116,117^

### 5.4. Alternatives to Factor XIII Concentrates

When specific FXIII concentrates are unavailable or cannot be procured rapidly, cryoprecipitate and plasma (fresh frozen plasma [FFP] or plasma frozen within 24 hours after phlebotomy [PF-24]) can serve as temporary bridges to raise FXIII activity, but they are second-line because of FXIII content variability, larger volumes (plasma), and lack of pathogen reduction in many settings.^119,120^ Quantitative studies show that FXIII content is highly variable between units and between products, underscoring the difficulty of precise dose-effect predictions.^121^ In practice, adult dosing often mirrors general factor replacement strategies (plasma [e.g., 10–20 mL/kg] or cryoprecipitate [e.g., 5–10 units / 1-2 pools in adults, roughly 1 unit/10 kg]),^122^ followed by FXIII activity testing to verify that hemostatic targets have been achieved and sustained over the procedural or bleeding risk interval. In resource-limited contexts, small scheduled volumes of plasma have been used as interim prophylaxis leveraging the long FXIII half-life, but such strategies are inferior to concentrate-based prophylaxis, less predictable, and more burdensome from a transfusion medicine standpoint; they should be regarded as temporizing measures while definitive therapy is arranged. Plasma-based products also bring transfusion risks (e.g., volume overload, allergic reactions) that must be balanced against the urgency of FXIII repletion.^123^ Overall, pdFXIII or recombinant FXIII remains the standard of care for prophylaxis and peri-operative support; cryoprecipitate/plasma should be reserved for bridging when concentrates are not immediately available, with dosing confirmed by FXIII activity and careful clinical reassessment.

In addition to plasma and cryoprecipitate, fibrinogen concentrates (e.g., Fibryga, RiaSTAP) may contain variable amounts of FXIII (product- and lot-dependent) and can offer unpredictable, incidental supplementation during bleeding when fibrinogen repletion is indicated.^124,125^ However, fibrinogen concentrates are not indicated as primary FXIII replacement, given the inconsistent FXIII content, lack of dosing targets, and inability to reliably achieve FXIII activity goals.

### 5.5. Antithrombotic Therapy in FXIII Deficiency

Scenarios may arise in which anticoagulation is indicated despite FXIII deficiency. Limited case-based experience suggests that direct oral anticoagulants (DOACs) can be administered alongside FXIII replacement without excess bleeding when prophylaxis maintains hemostatic troughs, but such decisions require individualized risk–benefit evaluation and close monitoring. In a 2024 case, rivaroxaban was given with pdFXIII while FXIII troughs were targeted at ~50% during DOAC loading, ~30% during maintenance, and ~20% for long-term prophylaxis, with no bleeding complications reported.^126^ As data remain sparse, shared decision-making and documentation of patient preferences are essential.

### 5.6. Global Access and Implementation Gaps

In many health systems, even in high-income regions, FXIII concentrate access remains uneven due to formulary limitations, supply interruptions, and cost constraints. Pragmatically, programs may rely on plasma or cryoprecipitate as solutions, though in lower-resource settings, the availability of these products is also variable.^119^ Context-specific algorithms that define when plasma/cryoprecipitate can serve as temporary bridges, establish pathways to procure concentrates, and incorporate assays for diagnosis and monitoring are critical for patient care while longer-term supply solutions are pursued.

## 6. New Findings and Future Directions

In this review, we emphasize several developments that have refined both diagnosis and management of patients with FXIII deficiency. International and national series have expanded the phenotype, including inhibitor cohorts that clarify associations, treatment responses, and relapse risk, while newer reports underscore morbidity among some heterozygotes. On the laboratory side, increasing adoption of quantitative ammonia release activity assays has been accompanied by recognition of testing pitfalls necessitating plasma blanks, while fluorogenic isopeptidase methods are entering clinical use. Together, these trends have improved diagnostic accuracy and therapeutic monitoring capabilities. Clinically, activity-guided targets remain dictated primarily by observational evidence informing pragmatic goals. Finally, contemporary guidance documents and trauma/critical-care literature now explicitly recognize acquired FXIII deficiency as a potential contributor to bleeding in surgery, trauma, and critical illness, prompting greater attention to targeted replacement and assay availability.^81,127,128^

Despite these advancements, sustained progress in FXIII deficiency will require studies that link target activity thresholds to outcomes in the settings where decisions are most time sensitive. Comparison of protocolized perioperative targets in surgery is critical—for example maintaining FXIII activity ≥30% versus ≥50% during major operations—against usual care, while capturing standardized outcomes (e.g., major bleeding, re-operation for hemostasis, transfusion exposure, thromboembolism, length of stay, cost). Pharmacokinetic/pharmacodynamic sub-studies are essential to define recovery and clearance across ages and comorbid states (e.g., inflammation, liver disease, cardiopulmonary bypass). In obstetrics, trimester-specific trough targets warrant prospective evaluation. Given the distinct challenges of immune-mediated deficiency, an international registry using harmonized definitions should evaluate immunosuppression strategies, time to remission and relapse, responsiveness to concentrate, and a standardized inhibitor titer.

Equally important are laboratory advances that shorten diagnostic delays and enable real-time dosing decisions. Assessment of ammonia release, isopeptidase, and amine-incorporation methods using shared reference plasmas and proficiency testing should focus on the clinically critical low activity range. In parallel, point-of-care assays deserve evaluation; for example, viscoelastic testing that incorporates a standardized fibrinolytic challenge to reveal fibrin cross-linking defects.

Finally, implementation science and policy will determine whether evidence translates into improved patient outcomes. Clinical trials and multicenter registries should evaluate and track real-world prophylaxis practices, surgical protocols, and clinical outcomes to inform evidence-based guidelines. For example, the SWIss Factor XIII Trial (SWIFT) trial was initiated in 2024 to assess whether repletion of FXIII improves outcomes in women with postpartum hemorrhage.^129^ In addition to the pending results from these studies, efforts to build diagnostic capacity in underserved regions, develop national formularies that ensure consistent concentrate availability, and create educational programs for clinicians and laboratory professionals are essential to closing the gap between what we know and what we deliver to patients with FXIII deficiency worldwide.

## 7. Conclusions

FXIII deficiency is a rare bleeding disorder that remains underdiagnosed because traditional coagulation tests are normal while the clinical phenotype may be subtle and delayed. Modern practice should move to quantitative FXIII activity measurement when the bleeding pattern is suspicious; antigenic phenotyping, molecular analysis, and inhibitor evaluation should be performed, when indicated. Prophylaxis with FXIII concentrates is effective and should be individualized to trough targets and clinical context, while perioperative and obstetric management benefits from activity-guided dosing. Awareness of analytic nuances and method harmonization improves the reliability of monitoring and, ultimately, patient outcomes.

### Statements and Declarations

The authors did not receive support from any organization for the submitted work. None of the authors have disclosures related to this article. Unrelated, JWJ reports research funding from Bayer and honorarium from Instrumentation Laboratories.

### Author Contributions

JWJ performed the literature search and data analysis and drafted the manuscript. GSB, VC, CAFV, BNS, and BDA critically revised the work. All authors approved the final version for submission.
